# Cancer‐testis gene *STK31* is regulated by methylation and promotes the development of pancreatic cancer

**DOI:** 10.1002/cam4.5472

**Published:** 2022-11-24

**Authors:** Hao Gao, Baobao Cai, Zipeng Lu, Guangfu Wang, Yong Gao, Yi Miao, Kuirong Jiang, Kai Zhang

**Affiliations:** ^1^ Pancreas Center the First Affiliated Hospital of Nanjing Medical University Nanjing China; ^2^ Pancreas Institution of Nanjing Medical University Nanjing China

**Keywords:** CT gene, immunotherapy, invasion, migration, proliferation, *STK31*

## Abstract

**Backgroud:**

Pancreatic cancer (PC) is a highly invasive malignancy with extremely poor prognosis. *STK31* has been identified as a cancer‐testis (CT) gene, but its function in PC has not been elucidated well.

**Methods:**

The effect of *STK31* on cell proliferation, migration and invasion was investigated by in vitro and in vivo experiments and total RNA sequencing and targeted bisulfite sequencing was applied to explore the potential regulatory mechanisms of *STK31* in PC.

**Results:**

By analysis of tissue samples and the clinicopathologic features, we found that *STK31* was reactivated in PC and associated with poor prognosis. In addition, the vitro and vivo studies indicated that *STK31* could promote PC progression by facilitating cell proliferation, migration and invasion, and the indication. Targeted Bisulfite Sequencing showed that *STK31* was regulated by methylation. Furthermore, the results of total RNA sequencing suggested that *STK31* was closely related to signal transduction, metabolism, and the immune system.

**Conclusions:**

This study demonstrates that *STK31*, as a CT gene, can promote the development of PC and is regulated by methylation. *STK31* could be considered as a potential therapeutic target for PC.

## Graphical abstract

STK31 was reactivated in PC and associated with poor prognosis. STK31 could promote PC progression by facilitating cell proliferation, migration and invasion in vitro and vivo studies. Targeted Bisulfite Sequencing showed that STK31 was regulated by methylation. And total RNA sequencing suggested that STK31 was closely related to signal transduction, metabolism and the immune system.

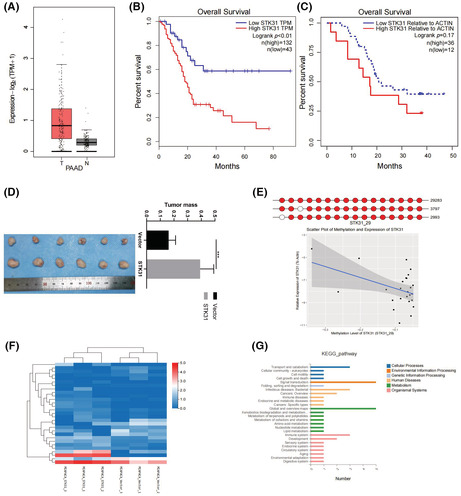

## 1 INTRODUCTION

Pancreatic cancer (PC) is a highly invasive malignancy and the prognosis is extremely poor. In the United States, it is the fourth main cause of cancer‐related deaths both in male and female with a poor 5‐year survival rate of 10%.^1^ Radical resection combined with systematic chemotherapy could be the only chance for curing the condition.^2^ And it seems promising that immunotherapy can benefit PC patients in clinical research.^3^ According to Oliver's study,^4^ radical resection combined with chemotherapy can provide a 5‐year survival rate to 30%–40%. However, only about 10%–15% of the patients have resectable lesions and half of PC patients present distant metastasis when diagnosed.^5^ Due to the high lethality and slow progression in diagnosing and treatment, PC is predicted to become the second leading cancer‐related mortality by 2030.^6^ Therefore, early diagnosis and new effective therapeutic targets would be the key to improving the outcomes of the condition in PC.

Cancer‐testis (CT) genes are a class of genes whose expression is restricted to testis in normal tissues, but frequently aberrantly expressed in some cancers.^7^ CT genes are considered as potential immunotherapy targets and diagnostic biomarkers of cancers owing to their fewer side effects because of the specific expression features.^8^, ^9^ Serine/threonine kinase 31 (*STK31*, also known as *TDRD8*) has been identified as a CT gene in PC in our previous study and we have elucidated the potential clinical value of this gene.^10^ In addition, *STK31* has also been detected in gastric cancer,^11^ colorectal cancer,^12^ cervical cancer,^12^ and lung cancer.^13^ The biological functions of *STK31* such as maintaining the undifferentiated state,^14^ promoting cell proliferation, and cell cycle regulation, has been studied in regards to these conditions.^15^ It's been found in our previous studies that the high expression of *STK31* suggested a poor prognosis of PC.^16^ However, the function of *STK31* in PC has not been demonstrated before. Thus, the aim of this present study was to elucidate the biological function and underlying mechanism of *STK31* in PC by in vivo and in vitro experiments, which could give us a better understanding of the development of PC and also offer a new possibility for targeted therapy.

## 2 MATERIAL AND METHODS

### 2.1 Patients and samples

Forty‐eight patients, who were confirmed as PC by two pathologists, were enrolled from the Pancreas Center, the First Affiliated Hospital with Nanjing Medical University. All these patients were underwent initial surgical resection between December 2016 and April 2017. In regards to public databases, the expression pattern can be confirmed by GEPIA2, which is a web service for analyzing the RNA sequencing expression data from the TCGA or GTEx projects by a standard processing pipeline.^17^ This study was approved by the Institutional Review Board of the First Affiliated Hospital with Nanjing Medical University (2021‐SR‐483) and a waiver of written informed consent was granted by the Institutional Review Board Committee.

### 2.2 Cell lines

Human PC cell lines BXPC‐3, Capan‐2, CFPAC‐1, COLO‐357, MIA PaCa‐2, and PANC‐1 were purchased from the Shanghai Cell Bank. The cell line hTERT‐HPNE was obtained from the American Type Culture Collection (ATCC, CRL‐4037). All these cell lines were cultured according to their manufacturer's instructions.

### 2.3 Transfection of siRNAs and plasmids

The small interfering RNAs (siRNAs) and plasmid to *STK31* were purchased from GenePharma (Shanghai, China) and GeneChem (Shanghai, China), respectively. The sequences of siRNAs were as follows: siRNA#1: 5’‐GGACCAG AAACUGAUUGAATT‐3′(sense) and 5’‐UUCAAUCAGU UUCUGGUCCTT‐3′ (antisense); siRNA#2: 5’‐GAGGAG UUCACCAGUGUUATT‐3′ (sense) and 5’‐UAACACUGG UGAACUCCUCTT‐3′ (antisense); siRNA#3: 5’‐GGAGA UAGCUCUGGUUGAUTT‐3′ (sense) and 5’‐AUCAACCA GAGCUAUCUCCTT‐3′ (antisense). The plasmid vector is GV144 (CMV‐EGFP‐MCS‐SV40‐Neomycin) and carrying the sequences of *STK31* (NM_032944). The transfection of siRNAs and plasmids were performed with Lipofectamine3000 according to the manufacturer's instructions. Cells were collected for subsequent experiments after transfection for 48–72 hours.

### 2.4 Preparation of STK31‐overexpressing cells

STK31‐overpressing lentivirus were constructed by OBiO Technology Corp. The full‐length coding region of human *STK31* was subcloned into the pLenti‐EF1a‐P2A‐Puro‐CMV‐STK31. Stable cell lines were selected by culturing in complete medium containing 5 μg/mL puromycin. *STK31* expression was confirmed by qRT‐PCR and western blotting.

### 2.5 Quantitative RT‐PCR and western blotting

Total RNAs were extracted from cells and tumor tissues by Total RNA Kit I (Omega Bio‐tek Inc) and then reverse‐transcribed into cDNA with HiScript II qRT SuperMix (Vazyme Inc). The qRT‐PCR was performed by the StepOnePlus Real‐Time PCR System (Applied Biosystems) and the primers were the same as in the previous study.^10^ The total protein from cells was extracted using a RIPA lysis buffer. The extracted protein was mixed with a 5× SDS‐PAGE sample loading buffer and boiled.

### 2.6 Targeted bisulfite sequencing and methylation detection

Genomic DNA from 27 tumor tissue samples of 48 patients was extracted by AllPrep DNA/RNA Mini Kit (Qiagen). Methylation analysis was performed with a working concentration of 20 ng/μL and 500 ng genomic DNA was subjected to bisulfite conversion using the EpiTect Fast DNA Bisulfite Kit (Qiagen). CpG sites located in the promoter of the *STK31* gene were selected from the 2 k upstream of a transcriptional start site (TSS) to the 1 k downstream of the first exon, and two CpG regions of the *STK31* gene were included. The methylation levels of the *STK31* gene promoter were analyzed by MethylTarget™ (Genesky Biotechnologies Inc.), an NGS‐based multiple methylation specific PCR analysis method. According to the manufacturer's protocol, the detection of *STK31* methylation was performed on Illumina Hiseq 2000 with a 2 × 150 bp sequencing mode. The methylation levels of the 33 CpG sites were measured.

### 2.7 Cell count kit‐8 assay and EdU assay

Cells transfected with siRNA and plasmids were seeded in 96‐well plates at a density of 30% and 70% area of per well. For cell count kit‐8 assay (CCK‐8), the CCK‐8 assay kit (Dojindo) was utilized to detect the ability of cell proliferation. The premixed 100 μl medium which consisted of 10:1 complete medium and CCK8 reagent was added to each well every 24 hours. After incubation in the dark at 37 °C for 2 hours, then the mixture was measured at 450 nm by a spectrometer. For EdU assay, a Cell Light EdU DNA imaging Kit (Ribo Bio) was used. Cell proliferation was evaluated by the proportion of positive stained cells. Three duplicated wells were detected.

### 2.8 Clone formation assay

The 1000 transfected cells were seeded in each well of 6‐well plate. The complete medium was changed every 5 days. After 10 days, the clones were stained with crystal violet solution. The number of clones was quantified to assess cell proliferation.

### 2.9 Cellular transwell assay

Twenty‐four well Millicell Hanging Cell Culture Inserts (Merck Millipore) and Biocoat Matrigel (BD Bioscience) were used to access the cell migration and invasion ability. 5 × 10^4^ transfected cells included in the 200 μl serum‐free medium were seeded in the upper chamber with an uncoated or Matrigel‐coated membrane, and 600 μl complete medium was added into the lower room as a chemoattractant. After 24 h of culturing for CFPAC‐1 and PANC‐1 and 48 h for MIA PaCa‐2, non‐migrating cells in the upper chamber were wiped. These cells migrating to the lower surface of the membrane were stained with crystal violet for 30 minutes and then photographed.

### 2.10 Animal study

Four‐week‐old male BALB/c nude mice were obtained from the Animal Center of the Nanjing Medical University. A total of 12 mice were randomly divided into stable *STK31*‐overexpressing and control MIA PaCa‐2 cells groups. 2 × 10^7^ tumor cells were injected into the axilla of mice for subcutaneous tumor formation. Once visible, the tumor size of these subcutaneous tumors were measured every 4 days. Four weeks later, after euthanizing all nude mice, the tumors were resected and photographed. And the size and weight of these subcutaneous tumor were measured. The animal use protocol has been reviewed and approved by the Animal Ethical and Welfare Committee (AEWC) of Nanjing Medical University (IACUC‐1904050).

### 2.11 RNA sequencing, differentially expressed genes (DGEs) identifying and function annotation and pathway enrichment analysis

RNA sequencing was performed by BGISEQ Genetic Sequencer (BGI, China). Stable MIA PaCa‐2 cells with overexpression of *STK31* was analyzed in comparison of vector control. Each group had three duplications. Genes with fold change (FC) ≥ 2 and adjusted *p* value≤0.001 were considered as DGEs. The gene ontology (GO) analysis and Kyoto Encyclopedia of Genes and Genomes (KEGG) analysis was performed for function annotation and pathway enrichment.

### 2.12 Statistical analysis

Statistical analysis was performed by Graphpad Prism (Version 8.0) and R (Version 4.1.1). Quantitative data were expressed as mean ± SD from three duplicate experiments. The difference of the mean between two groups was analyzed by Student's *t*‐test. All the data was representative of three independent repeated experiments. In both TCGA and our PC cohort, we considered those lower quartile expression levels as low expression and the rest as high expression. The correlation between *STK31* expression and methylation levels was examined by Pearson test. *p* < 0.05 was considered statistically significant.

## 3 RESULTS

### 3.1 Expression characterizations and roles of *STK31* in PC

**FIGURE 1:**
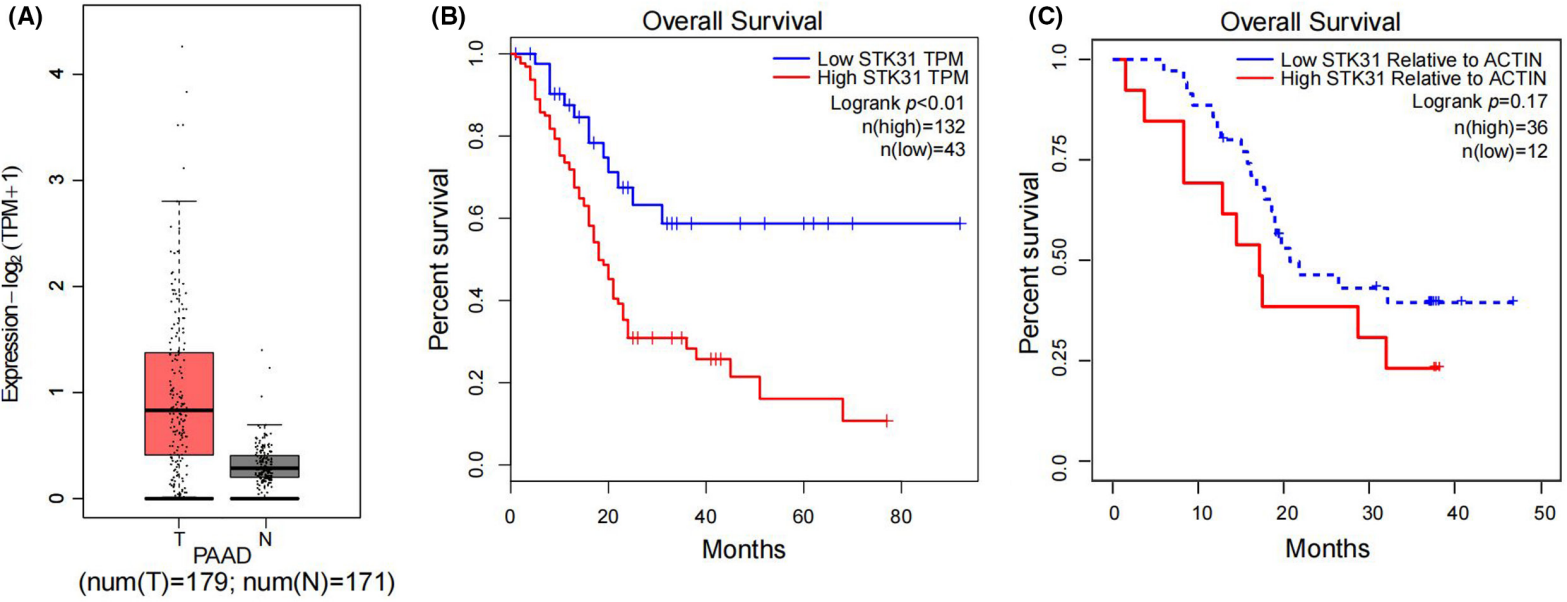
The expression pattern of STK31 gene. (A) STK31 was overexpressed in pancreatic cancer. Patients with relatively high expressions of STK31 had poorer overall survival rates in TCGA dataset (B) and samples from our biobank (C).

As we have found in previous studies, the *STK31* expression was restricted to the testis. We analyzed the expression of *STK31* in PC and examples of normal pancreas in GEPIA2, and it showed that the expression of *STK31* was higher in 179 PC than 171 normal pancreas (*p* < 0.05, Figure 1A). Furthermore, survival analysis revealed that patients with relatively high *STK31* expression had poorer overall survival (Figure 1B). The results from our biobank showed similar results (Figure 1C). This data suggested *STK31* was abnormally upregulated in PC and was associated with poor prognosis. The brief information of these PC patients from our cohort was described in Supplement Table 1.

### 3.2 Inhibition of *STK31* in CFPAC‐1 and PANC‐1 and overexpression in MIA PaCa‐2

**FIGURE 2:**
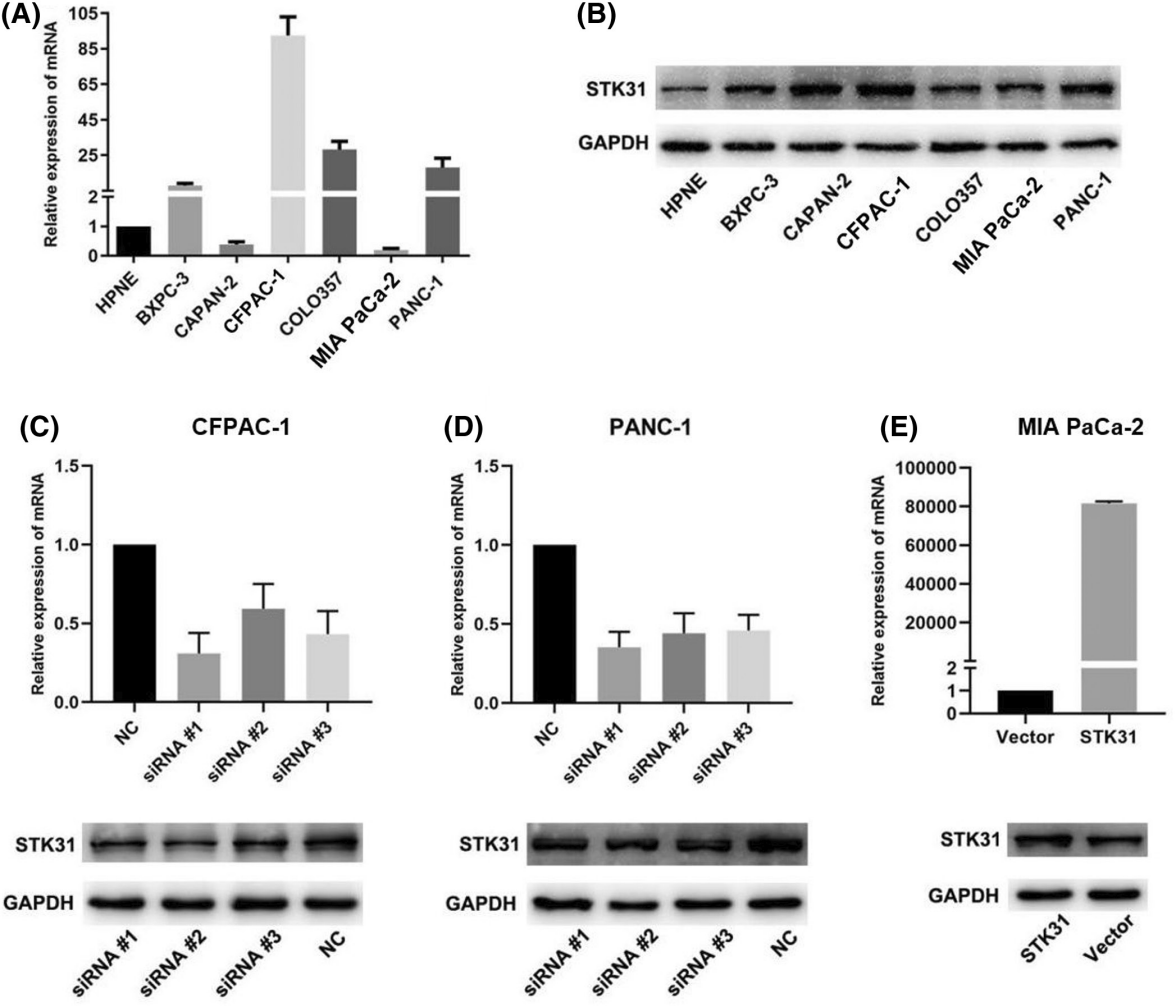
Knockdown and overexpression of STK31. The expression of STK31 was detected by qRT‐PCR (A) and western blot (B) in HPNE and PC cell lines. The interference efficiency for three siRNAs and plasmid of STK31 were examined by both qRT‐PCR and western blot in three PC cells—CFPAC‐1 (C), PANC‐1 (D), and MIA PaCa‐2 (E). The representative data of all experiments are presented as the mean ± SEM.

The expression of *STK31* was detected in hTERT‐HPNE cell line and six pancreatic cancer cell lines (Capan‐2, BXPC‐3, CFPAC‐1, MIA PaCa‐2, COLO‐357, and PANC‐1) by qRT‐PCR and western blot (Figure 2A, B). Compared to hTERT‐HPNE cell line, the *STK31* expression level of mRNA and protein was elevated in CFPAC‐1 and PANC‐1 while lower *STK31* expression was detected in MIA PaCa‐2. Hence, CFPAC‐1, PANC‐1, and MIA PaCa‐2 cell lines were selected for subsequent study. The interference efficiency for three siRNAs and plasmid of *STK31* were examined by both qRT‐PCR and western blot (Figure 1C–E). After evaluating the efficiency, siRNA #1 and the plasmid of *STK31* were applied for further functional experiments.

### 3.3 The effect of *STK31* on cell proliferation, migration, and invasion in PC

**FIGURE 3:**
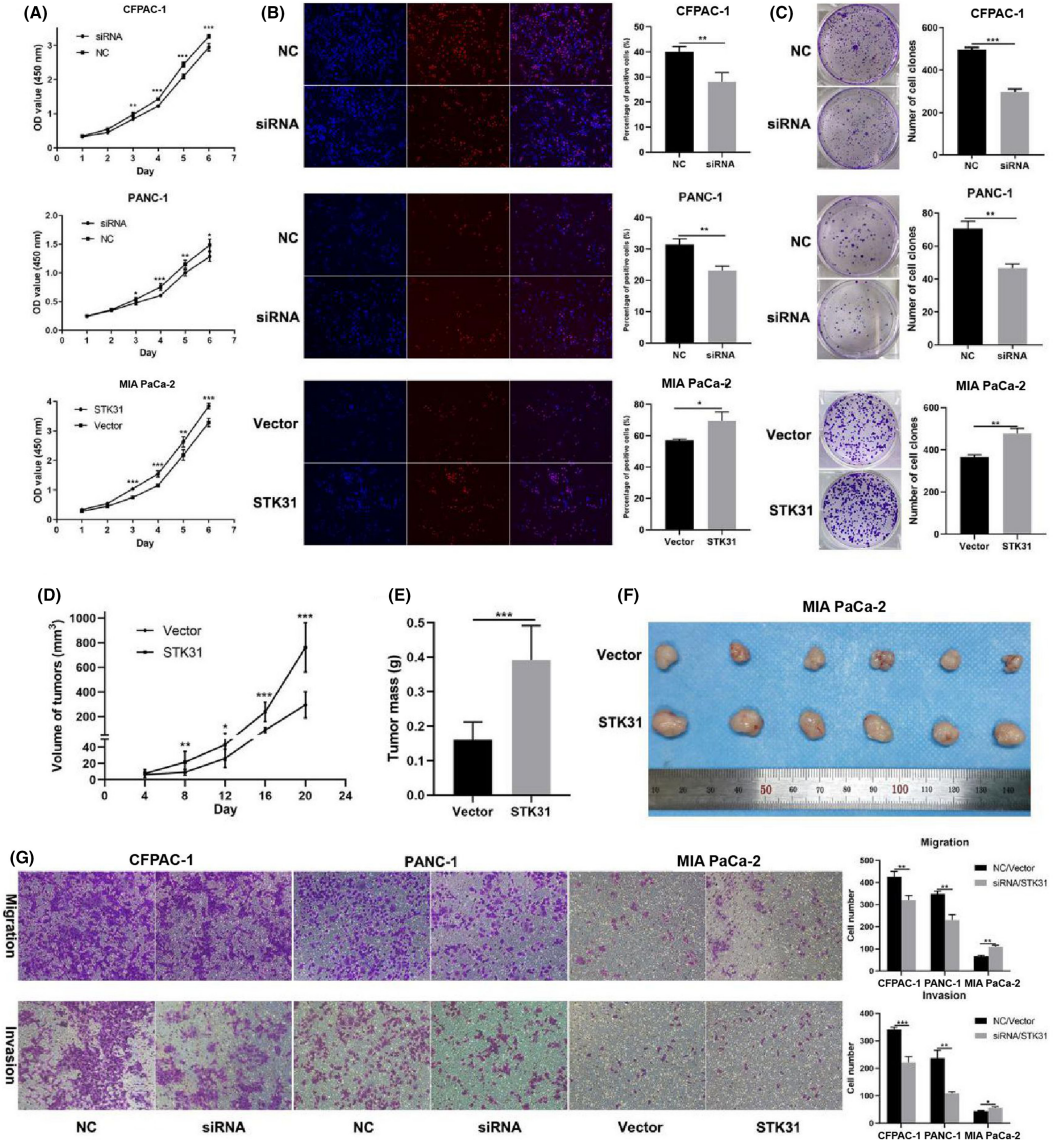
STK31 promoted cell proliferation, migration, and invasion in pancreatic cancer. CCK‐8 (A), EDU (B), and clone formation (C) were performed to investigate cell proliferation after STK31 was downregulated and overexpressed. Magnification for B: 100× and scale bar = 200 μm. Stable MIA PaCa‐2 cells with overexpressed STK31 and vectors were injected subcutaneously in nude mice. Tumors in mice injected with MIA PaCa‐2 cells overexpressing STK31 grew faster, as they had larger tumor volumes (D) and tumor mass (E & F). Cell migration and invasion were conducted, and the magnification is 100 × and scale bar = 100 μm (G). The representative data of all experiments are presented as the mean ± SEM. *p < 0.05, **p < 0.01 or ***p < 0.001.

CCK‐8, EDU, and clone formation assays were conducted to investigate the effect of *STK31* on pancreatic cancer cell lines. As shown in Figure 3A, the results of CCK‐8 demonstrated that the knockdown of *STK31* by siRNAs significantly impaired cell proliferation (*P* < 0.05). Similar results were obtained by EDU (Figure 3B) and clone formation assays (Figure 3C). On the contrary, overexpression of *STK31* in MIA PaCa‐2 promoted cell proliferation. To further determine the influence of *STK31* on PC cell proliferation, an in vivo study using stable *STK31*‐overexpressing MIA PaCa‐2 cell line prepared by *STK31*‐overexpressing lentivirus was performed. The results indicated that tumors in mice injected with overexpressing‐*STK31* MIA PaCa‐2 cells grew faster, as they had larger tumor volume and tumor mass (*p* < 0.05 in both comparisons) (Figure 3D–F). These results suggested that *STK31* could facilitate PC cell proliferation. The transwell assay was applied to investigate whether *STK31* has an effect on cell migration and invasion. As shown in Figure 3G, cell migration and invasion were inhibited after the downregulation of *STK31*, whereas enhanced results were observed following the overexpression of *STK31* (*p* < 0.05 in both comparisons). In conclusion, *STK31* could promote the ability of proliferation, migration, and invasion in PC cells.

### 3.4 The association between methylation and expression levels of *STK31*

**FIGURE 4:**
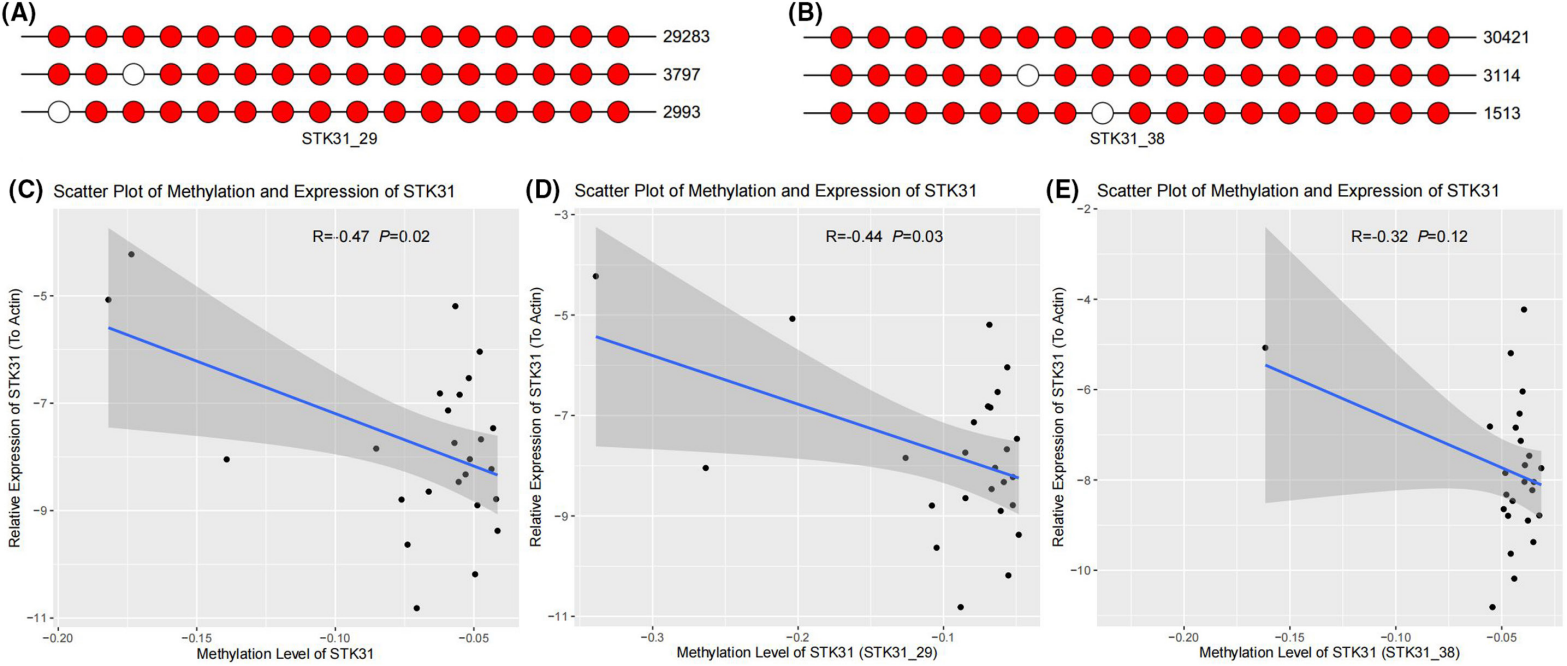
STK31 expression was regulated by methylation. Schematic diagrams showed the location of CpG sites STK31_29 (A) and STK31_38 (B). The visualizations of top three haplotype with the highest depth sequencing. The hollow circle represents unmethylated while the red circle represents methylated. There was an overall negative correlation between STK31 expression and methylation (C). The methylation level of STK31_29 (D) was negatively correlated with STK31 expression but STK31_38 (E) was not.

It is widely known that methylation could affect multiple physiological activities by regulating gene expression. To evaluate the effect of methylation on *STK31* expression, we applied MethylTarget to detect the methylation level in 27 pancreatic cancer tissue samples. Finally, two significant CpG sites (*STK31*_29: −277 to −123 of transcriptional start site and *STK31*_38: −105 to 77 of transcriptional start site) located at *STK31* were selected. Interestingly, the results showed that the methylation level of *STK31*_29 was negatively correlated with the expression of *STK31*, while *STK31*_38 was not (for *STK31*_29 CpG site: Pearson *r* = −0.44, *p* = 0.03; for *STK31*_38 CpG site: Pearson *r* = −0.32, *p* = 0.12; for total of two *STK31* CpG sites: Pearson *r* = −0.47, *p* = 0.02; Figure 4).

### 3.5 Identification of DGEs after overexpressing *STK31* and GO and KEGG pathway analysis of DGEs

**FIGURE 5:**
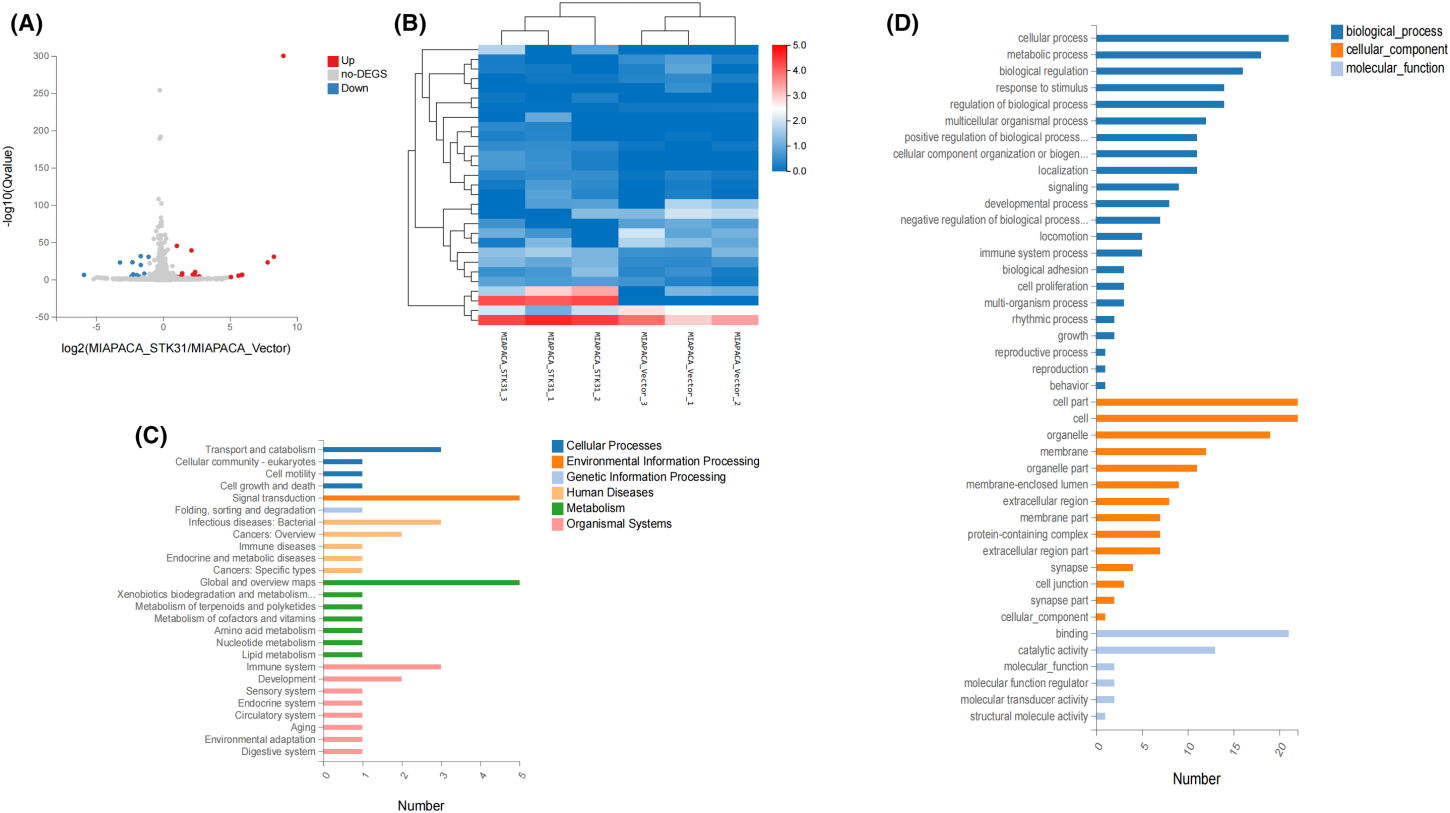
Exploration of biological functions of DEGs. (A) The volcano plot for DGEs comparing stable STK31‐ overexpressed MIA PaCa‐2 cells with corresponding control cells. Eighteen upregulated genes (red dots) and 11 downregulated genes (blue dots) were identified. (B) Heatmap of DGEs. (C) KEGG pathways analysis of these DGEs. (D) GO analysis of these DGEs

After overexpressing *STK31* in MIA PaCa‐2 cells, RNA sequencing was performed for further analysis. Twenty nine DGEs were identified including 18 upregulated and 11 downregulated genes (Figure 5A, B). To get further insight into the functions of the DGEs, GO and KEGG analyses were performed for these 29 genes. The KEGG analysis indicated that these DGEs were mainly associated with transport and catabolism, signal transduction, metabolism, immune system, and other biological pathways (Figure 5C). In GO biological process analysis, 29 DGEs were enriched in the cellular process, metabolic process as well as other biological processes (Figure 5D).

## 4 DISCUSSION

PC is a highly invasive and lethal malignancy with a poor prognosis. The complicated mechanism of tumorigenesis and its extremely malignant biological behaviour have made progression in early diagnosis and effective treatment stagnant.^2^, ^4^ In this study, the results indicated that *STK31* was abnormally reactivated in PC tissues. The high expression of *STK31* was associated with poor prognosis of PC. *STK31* could promote cancer progression by facilitating cell proliferation, migration, and invasion. As a result, *STK31* is a oncogene and can be considered as a potential therapeutic target for the condition.

*STK31* was first identified in the spermatogonia in mice, and the human ortholog *STK31* was also only detected in human testis among these samples from different parts of the body.^18^, ^19^ Then Wu et al.^20^ demonstrated *STK31* was detected in germ cells of human testis and located in the cytoplasm. Studies investigating *STK31* in cancers mostly focus on colorectal cancers before. In 2008, *STK31* was detected by laser microdissection and cDNA microarray in colorectal cancer cells.^12^ Furthermore, a STK31 peptide was able to induce immune responses by eliciting specific cytotoxic T lymphocytes.^12^ *STK31* was also reported to play a critical role to maintain the undifferentiated state of colon cancer cells, and a downregulation of *STK31* could significantly suppress cell proliferation both in vitro and vivo studies.^14^ Kuo et al. also indicated that the expression level of *STK31* in colorectal cancer cells was cell cycle‐dependent and that the abnormal overexpression of *STK31* enhanced the cell abilities of migration and invasion without altering cell proliferation.^15^ Furthermore, *STK31* was reported to be a potential biomarker in predicting the metastasis of colorectal cancer^21^ and to differentiate colorectal cancer from benign polyps.^22^ Except for colorectal cancer, *STK31* is also abnormally expressed in lung cancer. *STK31* was reported to promote cell proliferation in lung cancer cells by the Wnt/β‐catenin signaling pathway and positive feedback regulation of c‐myc.^13^ In general, the oncogenic role of *STK31* indicated by the present study was consistent with previous reports. However, some results in this study should be taken carefully. The ability of the MIA PaCa‐2 cell line to pass through well is relatively weak so we have to double the duration of cellular transwell assay of MIA PaCa‐2 cells. Moreover, we actually employed PANC‐1 and MIA PaCa‐2 cell lines in vivo to explore the effect of *STK31* on cell proliferation. However, subcutaneous tumorigenesis was barely observed in mice injected with PANC‐1 cells, and as a result the corresponding outcome was absent.

Previous studies have identified that *KRAS*, *CDKN2A*, *TP53*, and *SMAD4* was the major four mutation driver genes for PC.^2^ However, mutation driver genes can only explain part of the carcinogenesis. Epigenetic drivers are also identified as a significant characteristic of tumorigenesis.^23^ CT genes characterizing distinct expression in testis and some certain tumors are crucial members of epigenetic drivers.^24^ In addition to contributing to spermatogenesis,^25^, ^26^ *STK31* is frequently expressed in various cancers,^12^ thus serving as a CT gene. In our previous study, methylation is considered to be one of the mechanisms that regulates *STK31* expression through TCGA database analysis.^10^ In this study, we confirmed this result in the tumor tissues from our center. However, it is interesting that not all methylation levels of *SKT31* CpG sites were associated with the *STK31* expression. We found that only the *SKT31*_29 CpG site was associated with gene expression but not *STK31*_38.

Currently the biological functions of CT genes are mostly unclear. Emerging evidence suggested that they could contribute to tumorigenesis by regulating transcriptional activity, mitotic fidelity, and protein degradation.^9^ Due to the immune‐privileged status of the testis, ectopic expression of CT genes in cancers can induce immune responses with fewer side effects. As a result, CT genes products are also named CT antigens. The immunogenicity of CT antigens enables them to become potential targets for immunotherapy.^7^ MAGEA‐3 is a founding member of CT antigens and vaccines targeting MAGEA‐3 have been tested in patients.^27^ The safety and immunogenicity have been demonstrated in MAGEA‐3 positive patients with non‐small cell lung cancer.^28^ Another famous CT antigen, NY‐ESO‐1, has also initiated clinical trials. Preliminary results have shown clinically meaningful benefits, but the true effect needs further investigation.^29^, ^30^ JQ‐1, an inhibitor of the CT antigen *BRDT*, was reported to inhibit the growth of xenograft models in PC.^31^ Given that *STK31* was one of the CT genes and the RNA sequencing results in this study suggest that *STK31* was closely related to signal transduction and immune systems, it might be a potential target for immunotherapy of PC. In summary, although the safety and feasibility have been demonstrated, the true benefit of CT antigen‐based immunotherapy still needs further investigation. CT antigen specific immunotherapy in combination with chemotherapy or other immunotherapeutic treatments, such as checkpoint blockades, may be a promising direction.

Several limitations still existed in this study. First, although we have observed that *STK31* was an important effect on the development of PC, no in‐depth mechanism has been studied. We will investigate the mechanism of *STK31* affecting on PC in future studies. Second, we do not have a good way to affect the expression of STK31 in vivo to improve the prognosis of PC. Methylation, as well as miRNAs which were described in our previous studies,^16^ would be a potential way of treatment. Third, because of the limited sample size, we have not been able to definite a value of *STK31* expression level to distinguish the prognosis of PC, which is helpful for clinical decision making.

In conclusion, *STK31* was aberrantly upregulated and suggested a poor prognosis in patients with PC. *STK31* promotes development of PC by accelerating cell proliferation, migration, and invasion. However, the expression level of *STK31* is regulated by methylation. Due to the special CT gene expression pattern, *STK31* could be considered as a promising biomarker for early diagnosis and a hopeful therapeutic target for PC.

## AUTHOR CONTRIBUTIONS

**Hao Gao:** Data curation (equal); formal analysis (equal); writing – original draft (equal). **Baobao Cai:** Data curation (equal); formal analysis (equal); writing – original draft (equal). **Zipeng Lu:** Resources (supporting). **Guangfu Wang:** Data curation (supporting). **Yong Gao:** Data curation (supporting). **Yi Miao:** Project administration (supporting); supervision (equal). **Kuirong Jiang:** Conceptualization (supporting); project administration (equal); supervision (lead). **Kai Zhang:** Conceptualization (lead); resources (equal); validation (lead); writing – review and editing (lead).

## CONFLICTS OF INTEREST

The authors have no conflict of interest to declare .

## ETHICAL APPROVAL

This study was approved by the Institutional Review Board of the First Affiliated Hospital of Nanjing Medical University (2021‐SR‐483).

## Supporting information


Table S1

## Data Availability

Not available.
