# Supplementary material for: Cancer‐testis gene STK31 is regulated by methylation and promotes the development of pancreatic cancer
Source: Cancer Med. 2022 Nov 24;12(6):7273–82. doi: 10.1002/cam4.5472 (PMC10067059; doi:10.1002/cam4.5472)
Supplement: Supplementary file 1 — Table S1 [file CAM4-12-7273-s001.docx]

| Supplement Table 1. Clinical characteristics of patients (N=48) with PC from our Pancreas BioBank | |
| --- | --- |
|  | Characteristic |
| Age (y, Mean±SD.) | 61.4±10.7 |
| Gender N(%) |  |
| Male | 27 (56) |
| Female | 21 (44) |
| CA19-9 (U/ml, IQR) | 161.0（45.4-505.7） |
| T stage N(%) |  |
| T1 | 5 (10) |
| T2 | 31(65) |
| T3 | 4 (8) |
| T4 | 5 (10) |
| Unknown | 3 (6) |
| N stage N(%) |  |
| N0 | 24 (50) |
| N1/N2 | 24 (50) |
| M stage N(%) |  |
| M0 | 48 (100) |
| M1 | 0 (0) |
| TNM stage N(%) |  |
| I | 16 (33) |
| II | 18 (38) |
| III | 11 (23) |
| Unknown | 3 (6) |
| Grade N(%) |  |
| I/I-II/II | 10 (21) |
| II-III/III | 36 (75) |
| Unknown | 2 (4) |
